# ﻿Multi-locus molecular phylogenetic analysis reveals four new species and a new record of *Ophiocordyceps* (Ophiocordycipitaceae, Hypocreales) on dipteran hosts in Thailand

**DOI:** 10.3897/mycokeys.119.155439

**Published:** 2025-07-07

**Authors:** Suchada Mongkolsamrit, Donnaya Thanakitpipattana, Wasana Noisripoom, Kanoksri Tasanathai, Kanraya Liangsiri, Somruetai Jaiyen, Nattawut Rungjindamai, Marc Stadler, Jennifer Luangsa-ard

**Affiliations:** 1 National Center for Genetic Engineering and Biotechnology (BIOTEC), National Science and Technology Development Agency (NSTDA), 111 Thailand Science Park, Phahonyothin Road, Khlong Nueng, Khlong Luang, Pathum Thani, 12120, Thailand National Center for Genetic Engineering and Biotechnology Pathum Thani Thailand; 2 Department of Biology, Faculty of Science, King Mongkut’s Institute of Technology Ladkrabang (KMITL), Chalongkrung Road, Ladkrabang, Bangkok, 10520, Thailand King Mongkut’s Institute of Technology Ladkrabang Bangkok Thailand; 3 Department of Microbial Drugs, Helmholtz Centre for Infection Research GmbH (HZI), Inhoffenstraße 7, 38124 Braunschweig, Germany Helmholtz Centre for Infection Research GmbH (HZI) Braunschweig Germany

**Keywords:** cryptic species, entomogenous fungi, *Hymenostilbe*, new taxa, phylogenetic analyses

## Abstract

During field surveys conducted in various regions of Thailand, several fungal specimens parasitising flies were discovered. These fungi exhibited morphological characteristics consistent with the broad concept of *Ophiocordycepsdipterigena*, including yellow to orange-brown cylindrical stromata bearing fertile ascomata at the tip. Multi-locus phylogenetic analyses based on ITS, LSU, *TEF1*, and *RPB2* sequences revealed that these specimens belong to a well-supported monophyletic clade, referred to as the '*O.dipterigena*' complex, which forms a distinct subclade within the hymenostilboid clade. This complex comprises four newly described species with clearly defined host associations: *O.floriformis*, found on robber flies (Asilidae), particularly on *Clephydroneura* sp.; *O.muscae*, isolated from the housefly (*Muscadomestica*); *O.tabani*, parasitising horse flies (*Tabanus* sp.); and *O.thilosuensis*, infecting fruit flies (*Anastrephaobliqua*) and soldier flies (Sarginae). Additionally, several strains clustered with the previously described *O.philippinensis*, which was also collected from *Clephydroneura* sp. This marks the first record of *O.philippinensis* in Thailand.

## ﻿Introduction

The genus *Ophiocordyceps*Petch (1931) represents an entomopathogenic fungus of considerable ecological and economic importance. For instance, *Ophiocordycepssinensis* is acknowledged as a valuable source of medicinal compounds (Chellapandi and Saranya 2024; Huang et al. 2024). It is the largest genus in the family Ophiocordycipitaceae within the order Hypocreales and is typified by *Ophiocordycepsblattae* Petch, which infects cockroaches (Blaberidae, Blattodea). Species in this genus parasitise insects from various orders, including Coleoptera, Diptera, Hemiptera, Hymenoptera, Lepidoptera, Odonata, and Orthoptera, targeting all life stages, such as larvae, pupae, nymphs, and adults (Luangsa-ard et al. 2018; Khonsanit et al. 2019; Tasanathai et al. 2019, 2020; Fan et al. 2021; Zha et al. 2021). Species in *Ophiocordyceps* exhibit a wide variety of morphological characteristics, ranging from soft, tough, and dark stromata to brightly coloured ones, spanning shades from yellow to red. The perithecia associated with these species can either be completely embedded or superficial. Their ascospores are filiform, entire with multiple septa, or fragmented into part-spores. The predominant asexual morphs associated with *Ophiocordyceps* are Hirsutella-like, followed by Hymenostilbe-like, Syngliocladium-like, and Stilbella-like (Khonsanit et al. 2019; Tasanathai et al. 2019; Mongkolsamrit et al. 2023, 2024). They can be found inhabiting insect hosts buried in soil, on fallen leaves, decaying wood, the underside of leaves, and on stems of forest plants. The position, shape, and colour of the fertile parts of the stromata are important morphological characteristics essential for species identification.

Many species within *Ophiocordyceps* share overlapping morphological features, making it challenging to distinguish individual species based on morphology alone. Recent studies have highlighted the known diversity of *Ophiocordyceps*, with new species being described from various hosts, including ants (e.g., *O.acroasca*, *O.ansiformis*, *O.basiasca*, *O.bifertilis*, *O.contiispora*, *O.laotii*, *O.nuozhaduensis*, *O.subtiliphialida*, and *O.tortuosa*) (Mongkolsamrit et al. 2023; Tang et al. 2023a, b); Coleoptera (e.g., *O.borealis*, *O.brunnea*, *O.capilliformis*, *O.kohchangensis*, *O.phitsanulokensis*, *O.pseudovariabilis*, and *O.ratchaburiensis*) (Zha et al. 2021; Mongkolsamrit et al. 2024); and Lepidoptera (e.g., *O.fenggangensis*, *O.alboperitheciata*, *O.campes*, *O.longistromata*, *O.musicaudata*, and *O.phuwiangensis*) (Tasanathai et al. 2020; Peng et al. 2024); Orthoptera (e.g., *O.kobayasii*, *O.krachonicola*) (Thanakitpipattana et al. 2020); stink bugs (Hemiptera) (e.g., *O.asiana*, *O.poecilometigena*, *O.tessaratomidarum*) (Khao-ngam et al. 2021; Crous et al. 2023); leafhoppers (Hemiptera) (e.g., *O.flavida*) (Mongkolsamrit et al. 2021); and termites (e.g., *O.fusiformis*, *O.globiperitheciata*) (Tasanathai et al. 2022; Fan et al. 2024). Most species have been described based on micro- and macromorphology, along with phylogenetic analyses, which have significantly enhanced the accuracy of species identification and classification, resulting in a notable increase in the number of *Ophiocordyceps* species recognised. Although species in *Ophiocordyceps* have been reported to infect insects in various orders, they primarily target Coleoptera, Hymenoptera, and Lepidoptera.

Diptera, or true flies, play essential roles in ecosystems as pollinators, decomposers, and key components of food webs. Some species, such as fruit flies (Tephritidae), are agricultural pests (Hudiwaku et al. 2021; Scolari et al. 2021), while others, including *Anagonialasiophthalma*, *Exoristasegregata*, and *Pentatomophagalatifascia* (Tachinidae), contribute to natural pest control (Chen et al. 2020; Hammami et al. 2023; Martins et al. 2023). Additionally, Diptera includes important disease vectors, such as mosquitoes, and research models such as *Drosophilamelanogaster* (Nainu et al. 2022; Mead et al. 2024), highlighting their significance for ecosystems and pest management. Entomopathogenic fungi in the genus *Ophiocordyceps* that parasitise flies (Diptera) include *O.dipterigena* (Berk. & Broome) G.H. Sung, J.M. Sung, Hywel-Jones & Spatafora, first described from a specimen collected from an adult fly in Sri Lanka (Berkeley and Broome 1873). *Ophiocordycepsdipterigena* is characterised by its pale brown to brown stipe emerging from the thorax of the dipteran host. The fertile ascomata are located at the tip of the stipe. This species is commonly found in forests, where it typically attaches by mycelium to surfaces such as the undersides of leaves, stems, or twigs of plants. Petch (1932) reported that the asexual morph of *O.dipterigena* is *Hymenostilbedipterigena*. Later, *Ophiocordyceps* species associated with adult flies have been reported, including *O.discoideicapitata* from Japan (Kobayasi and Shimizu 1982), *O.lacrimoidis* and *O.muscicola* from Brazil (Freire 2015; Hyde et al. 2016), *O.globiceps* from Thailand (Xiao et al. 2019), and *O.philippinensis* from the Philippines (Crous et al. 2023).

Based on previous reports of several *Ophiocordyceps* species found on adult flies, our study in Thailand aims to further explore the diversity of pathogenic fungi infecting flies (Diptera). We examined fungal specimens initially identified as *Ophiocordycepsdipterigena* due to the limited knowledge about the relationship between these fungi and their insect hosts. The specimens were obtained from the BIOTEC Bangkok Herbarium (BBH) and the Thailand Bioresource Research Centre (TBRC), the National Centre for Genetic Engineering and Biotechnology. The primary goal of this study was to enhance our understanding of the diversity within this group. Using an integrative taxonomic approach, we discovered four new species and recorded *O.philippinensis* as a new addition. Each species is described in detail, including morphological and phylogenetic data, as well as the identification of their respective dipteran hosts.

## ﻿Materials and methods

### ﻿Collections and isolation

The fungal specimens were collected from various forests in Thailand, including those in Nakhon Ratchasima, Phetchabun, Chaiyaphum, and Tak provinces. We specifically searched for fungi occurring on dipterans attached to twigs and both the upper and underside of leaves on forest trees. The specimens were placed in plastic containers for transport to the laboratory for isolation. The materials were then examined under a dissecting microscope (Olympus SZ61). The protocol for isolating ascospores and conidia from the fertile parts followed methods from previous studies (Luangsa-ard et al. 2018; Mongkolsamrit et al. 2018), using potato dextrose agar (PDA) plates (PDA: freshly diced potato 200 g/L, dextrose 20 g/L, agar 15 g/L). PDA plates were checked for contamination and consistency before use. After overnight incubation at room temperature, the inoculated PDA medium was examined under a stereomicroscope to locate germinated ascospores and conidia. These germinated structures were transferred to fresh PDA plates and incubated for 30–35 days at 25 °C under light/dark conditions (L:D 14:10). The pure cultures were deposited at the Thailand Bioresource Research Centre (TBRC), National Centre for Genetic Engineering and Biotechnology, Thailand. All fungal specimens were dried overnight in an electric food dryer at 50–55 °C and subsequently deposited in the BIOTEC Bangkok Herbarium (BBH), National Biobank of Thailand.

### ﻿Host identification

Identification of dead dipteran hosts was conducted following the isolation of pure fungal cultures. The hosts were identified based on distinct morphological features of specific body regions, including the head (eyes, antennae, and mouthparts), thorax (legs and wings), and abdomen (ovipositor). Specimens were examined under a stereomicroscope.

### ﻿Morphological study

Macroscopic characteristics were observed from natural specimens, while microscopic features of perithecia, asci, ascospores, phialides, and conidia were examined on microscope slides mounted in lactophenol cotton blue. The shapes, sizes, and colours of these structures were measured and documented following the methods of Mongkolsamrit et al. (2020). Fungal strains were cultured on oatmeal agar (OA, Difco: oatmeal 60 g/L, agar 12.5 g/L) and PDA plates at 25 °C under light/dark cycles (L:D 14:10) for 30–35 days. Cultures were examined to compare morphological features, including phialides, conidia, and colony pigmentation. The colours of specimens and cultures grown on OA and PDA were described and standardised using the Royal Horticultural Society (RHS) Colour Chart (6^th^ Edition) (RHS 2015).

### ﻿DNA extraction, amplification, and sequencing

Genomic DNA was extracted from specimens or actively growing mycelial masses cultivated on PDA using a modified cetyltrimethylammonium bromide (CTAB) method (Doyle 1987). The fungal mycelium was carefully scraped from the agar surface with a sterile spatula and lysed in 600 μL of CTAB extraction buffer (1 M Tris-HCl, 5 M NaCl, 0.5 M EDTA, CTAB, PVP-40). The mycelium was then ground using a sterile pestle and incubated at 65 °C for 30 min. After incubation, 600 μL of chloroform:isoamyl alcohol (24:1) was added, and the mixture was gently inverted to mix. The tubes were centrifuged at 12,000 rpm for 15 min, and the upper aqueous phase was transferred to a fresh tube. DNA precipitation was performed by adding 300 μL of ice-cold isopropanol, followed by incubation at –20 °C for 1 h. The samples were centrifuged again at 4 °C at 12,000 rpm for 20 min to pellet the DNA. The resulting DNA pellet was washed with 70% ethanol and centrifuged at 12,000 rpm for another 20 min. Finally, the DNA pellets were air-dried, dissolved in 1× TE buffer, and stored at –20 °C until further use. Nuclear loci, including the rDNA region encompassing the internal transcribed spacer (ITS) regions ITS1 and ITS2, the 28S rDNA (LSU), the translation elongation factor 1-α gene (TEF1), and the second largest subunit of RNA polymerase II (RPB2), were amplified and sequenced. The primer pairs used were ITS5/ITS4 for ITS (White et al. 1990), LR0R/LR7 for LSU (Vilgalys and Hester 1990), EF1-983f/EF1-2218r for *TEF1* (Rehner and Buckley 2005), and RPB2-5F2/RPB2-7Cr for *RPB2* (Liu et al. 1999; O’Donnell et al. 2007). Amplification reactions were performed in 25 μL reaction volumes containing 1 × PCR buffer (20 mM Tris-HCl, 50 mM KCl), 2.5 mM MgCl_2_, 0.4 M betaine, 200 μM of each dNTP, 0.5 μM of each forward and reverse primer, 1 U Taq DNA polymerase (Thermo Scientific), and 50–100 ng of DNA template. Amplifications were conducted under the following conditions: for ITS and LSU, 2 min at 95 °C; 34 cycles of 1 min at 95 °C, 2 min at 55 °C, and 2.3 min at 72 °C; and a final extension of 10 min at 72 °C. For *TEF1*: 2 min at 95 °C; 34 cycles of 1 min at 95 °C, 1 min at 55 °C, and 2 min at 72 °C; with a final extension of 2 min at 72 °C. For *RPB2*: 3 min at 94 °C; 34 cycles of 1 min at 94 °C, 1 min at 50 °C, and 1.3 min at 72 °C; followed by a final extension of 8 min at 72 °C. Amplifications were performed using a Bio-Rad T100 thermal cycler (Bio-Rad Laboratories, Hercules, CA, USA). The purified PCR products were sequenced with the same primers used for amplification using the Sanger dideoxy method (Macrogen Inc., Seoul, South Korea).

### ﻿Phylogenetic analyses

The DNA sequences generated in this study were checked for ambiguous base calls using BioEdit v. 7.2.5. Unambiguous sequences were submitted to GenBank. Sequences of ITS, LSU, *TEF1*, and *RPB2* were compiled for alignment, including those from previous studies, as listed in Table 1. Sequence alignments were performed using Clustal W (Thompson et al. 1994) and manually edited in BioEdit. Phylogenetic analyses of the combined alignments were conducted using RAxML-HPC2 on XSEDE v. 8.2.12 (Stamatakis 2014) via the CIPRES Science Gateway portal, employing the GTRGAMMA+I model with 1,000 bootstrap iterations. Bayesian inference (BI) was performed in MrBayes v. 3.2.7a (Ronquist et al. 2012), with best-fit models selected using MrModeltest v. 2.2 (Nylander 2004). The optimal model was GTR+G+I. Markov chain Monte Carlo (MCMC) simulations were run for 5,000,000 generations, sampling every 1,000 generations and discarding the first 10% as burn-in. Bayesian posterior probabilities (PP) were calculated from the remaining trees. The RAxML and BI outputs were visualised using TreeView v. 1.6.6 (Page 1996).

**Table 1.** List of taxa included in the phylogenetic analysis and their GenBank accession numbers.

Species	Strain No.	Host/ Substratum	GenBank accession numbers				References
			ITS	LSU	*TEF1*	*RPB2*	
*Ophiocordycepsasiana*	BCC 84234^T^	Coreidae	MW285708	MW280201	MW292438	–	Khao-ngam et al. (2021)
*O.australis*	HUA 186147	Hymenoptera	KF937351	KC610764	KC610734	–	Sanjuan et al. (2015)
*O.australis*	HUA 186097	Hymenoptera	KF937350	KC610765	KC610735	–	Sanjuan et al. (2015)
*O.blattae*	BCC 34765	Blattodea	–	–	MT533484	–	Mongkolsamrit et al. (2021)
*O.blattae*	BCC 38241	Blattodea	–	MT512657	MT533485	–	Mongkolsamrit et al. (2021)
*O.brunneipunctata*	OSC 128576	Coleoptera	–	DQ518756	DQ522324	DQ522420	Spatafora et al. (2007)
*O.buquetii*	HMAS 199613	Hymenoptera	–	KJ878904	KJ878984	–	Quandt et al. (2014)
*O.buquetii*	HMAS 199617	–	–	KJ878905	KJ878985	–	Quandt et al. (2014)
*O.communis*	BCC 1842	Termitidae	MH754726	MH753680	MK284266	MK214096	Tasanathai et al. (2019)
*O.communis*	BCC 1874	Termitidae	MH754725	MH753679	MK284267	MK214095	Tasanathai et al. (2019)
*O.curculionum*	OSC 151910	Coleoptera	–	KJ878885	–	–	Quandt et al. (2014)
*O.dipterigena*	OSC 151911	Diptera	–	KJ878886	KJ878966	–	Quandt et al. (2014)
*O.dipterigena*	OSC 151912	Diptera	–	KJ878887	KJ878967	–	Quandt et al. (2014)
*O.evansii*	HUA 186159^T^	Hymenoptera	KP200889	KC610770	KC610736	–	Sanjuan et al. (2015)
*O.evansii*	HUA 186163	Hymenoptera	KP200890	KC610771	KC610737	–	Sanjuan et al. (2015)
***O.floriformis***	**BBH 27634**	**Diptera**	**PV170894**	**OP493200**	**OP503163**	**OP503164**	**This study**
***O.floriformis***	**BBH 51295**	**Diptera**	**PV170895**	**PV257643**	**PV274276**	**PV274287**	**This study**
*O.forquignonii*	OSC 151908	Diptera	–	KJ878889	–	KJ878947	Quandt et al. (2014)
*O.forquignonii*	OSC 151902	Diptera	–	KJ878876	–	KJ878945	Quandt et al. (2014)
*O.globiceps*	MFLUCC 18-0495	Diptera	MH725815	MH725829	MH727387	–	Xiao et al. 2019
*O.globiceps*	MFLU 18-0661^T^	Diptera	MH725816	MH725830	MH727388	–	Xiao et al. 2019
*O.granospora*	BCC 82255^T^	Hymenoptera	MH028143	MH028156	MH028183	MH028177	Khonsanit et al. (2019)
*O.granospora*	BCC 82256	Hymenoptera	MH028144	MH028157	–	MH028178	Khonsanit et al. (2019)
*O.hemisphaerica*	FLOR59525^T^	Diptera	KX197233	–	–	–	Hyde et al. (2016)
*O.hemisphaerica*	FLOR59542	Diptera	KX197234	–	–	–	Hyde et al. (2016)
*O.hemisphaerica*	FLOR59553	Diptera	KX197235	–	–	–	Hyde et al. (2016)
*O.houaynhangensis*	MY11460	Coleoptera	MH092892	MH092908	MH092899	–	Crous et al. (2018)
*O.houaynhangensis*	MY11461	Coleoptera	MH092893	MH092909	MH092900	–	Crous et al. (2018)
*O.irangiensis*	NBRC 101400	Hymenoptera	JN943335	JN941426	—	–	Schoch et al. (2012)
*O.khaoyaiensis*	BCC 82796^T^	Hymenoptera	MH028150	MH028153	MH028187	MH028175	Khonsanit et al. (2019)
*O.khaoyaiensis*	BCC 82797	Hymenoptera	MH028151	MH028154	MH028188	MH028176	Khonsanit et al. (2019)
*O.lacrimoidis*	FLOR 59552^T^	Diptera	KX197231	–	–	–	Hyde et al. (2016)
*O.longissima*	HMAS 199600	–	–	–	KJ878972	KJ878949	Quandt et al. (2014)
*O.longissima*	EFCC 6814	Hemiptera	–	EF468817	EF468757	–	Sung et al. (2007)
*O.megacuculla*	BCC 82262	Hymenoptera	MH028146	MH028161	MH028191	MH028180	Khonsanit et al. (2019)
*O.megacuculla*	BCC 82984^T^	Hymenoptera	MH028148	MH028162	MH028192	MH028181	Khonsanit et al. (2019)
***O.muscae***	**BCC 72871**	**Diptera**	–	**PV257644**	–	**PV274288**	**This study**
***O.muscae***	**BCC 73607**	**Diptera**	**PV170896**	**PV257645**	**PV274277**	**PV274289**	**This study**
***O.muscae***	**BCC 73616**	**Diptera**	**PV170897**	**PV257646**	**PV274278**	**PV274290**	**This study**
O.muscae	NHJ12170.02	Diptera	GU723771	–	GU797127	–	Luangsa-ard et al. (2011)
*O.muscae*	MRCIF71	Diptera	EU573346	–	–	–	Freire (2015)
*O.myrmecophila*	TN S 27120	Hymenoptera	–	KJ878895	KJ878975	–	Quandt et al. (2014)
*O.myrmecophila*	HMAS 199620	Hymenoptera	–	KJ878893	KJ878973	–	Quandt et al. (2014)
*O.nutans*	OSC 110994	Hemiptera	–	DQ518763	DQ522333	–	Spatafora et al. (2007)
*O.odonatae*	TNS F 27117	–	–	KJ878878	–	–	Quandt et al. (2014)
*O.donatae*	TNS F 18563	Odonata (Dragonfly)	AB104725	KJ878877	–	–	Quandt et al. (2014)
*O.philippinensis*	LOD PF 4565^T^	Diptera	OQ641807	OQ641808	OQ660303	–	Crous et al. (2023)
***O.philippinensis***	**BCC 79225**	**Diptera**	**PV170899**	**PV257648**	**PV274280**	–	**This study**
***O.philippinensis***	**BCC 78339**	**Diptera**	**PV170900**	**PV257649**	**PV274281**	–	**This study**
***O.philippinensis***	**BCC 22048**	**Diptera**	**PV170898**	**PV257647**	**PV274279**	**PV274291**	**This study**
***O.philippinensis***	**BCC 79871**	**Diptera**	–	–	**PV274282**	**PV274292**	**This study**
***O.philippinensis***	**BCC 79872**	**Diptera**	–	**PV257650**	**PV274283**	**PV274293**	**This study**
*O.sobolifera*	KEW 78842	Hemiptera	JN049855	EF468828	–	EF468925	Sung et al. (2007)
*O.sobolifera*	TNS F 18521	Hemiptera	–	KJ878898	KJ878979	–	Quandt et al. (2014)
*O.sphecocephala*	NBRC 101416	Hymenoptera	JN943348	JN941443	–	–	Schoch et al. (2012)
***O.tabani***	**BCC 45127**	**Diptera**	**PV170901**	**PV257652**	–	**PV339938**	**This study**
***O.tabani***	**BCC 39918**	**Diptera**	–	**PV257651**	**PV274284**	—	**This study**
*O.termiticola*	BCC 1920^T^	Termitidae	MH754724	MH753678	MK284265	MK214094	Tasanathai et al. (2019)
*O.termiticola*	BCC 1770	Termitidae	–	MH753677	MK284264	MK214093	Tasanathai et al. (2019)
*O.tessaratomidarum*	MY10830^T^	Tessaratomidae	–	MW280218	MW292434	–	Khao-ngam et al. (2021)
***O.thilosuensis***	**BCC 46607**	**Diptera**	**PV170903**	**PV257654**	**PV274286**	–	**This study**
***O.thilosuensis***	**BCC 47494**	**Diptera**	**PV170902**	**PV257653**	**PV274285**	**PV274294**	**This study**
*O.tricentri*	–	Hemiptera	AB027376	–	–	–	Nikoh et al. (2000)
*O.yakusimensis*	HMAS 199604	Hemiptera	–	KJ878902	–	KJ878953	Quandt et al. (2014)
*Paraisariagracilis*	EFCC 8572	Lepidoptera	JN049851	EF468811	EF468751	EF468912	Sung et al. (2007)
*P.gracilis*	EFCC 3101	Lepidoptera	–	EF468810	EF468750	EF468913	Sung et al. (2007)

^T^ex-type culture.

## ﻿Results

### ﻿Molecular phylogeny

**Figure 1.**
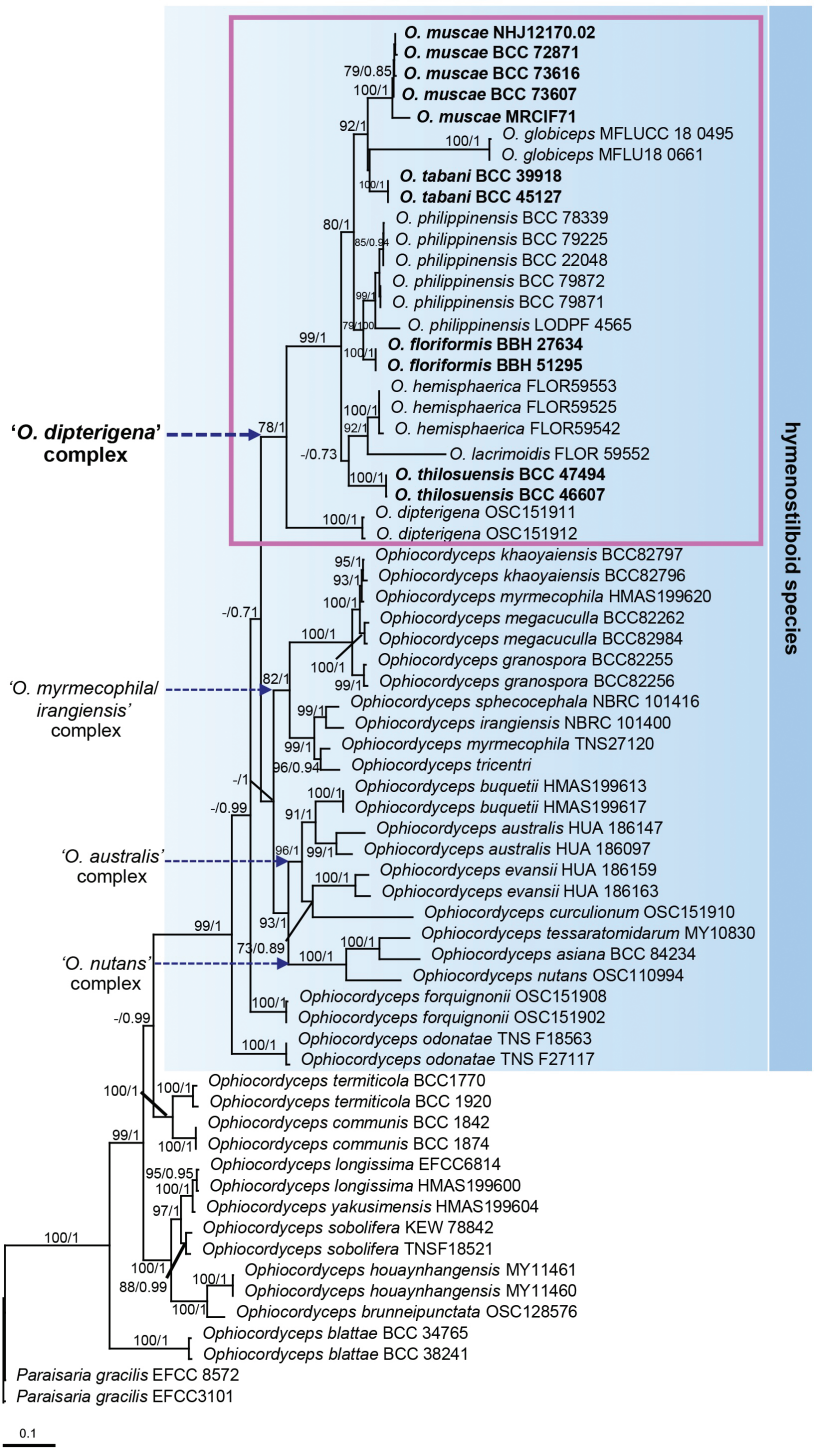
RAxML tree of the *Ophiocordycepsdipterigena* complex (highlighted in pink) and related species of entomopathogenic fungi in *Ophiocordyceps*, based on a combined ITS, LSU, *TEF1*, and *RPB2* dataset. Numbers at the major nodes indicate maximum likelihood bootstrap values (MLB ≥ 70%) and Bayesian posterior probabilities (BPP ≥ 0.70).

The ITS region, LSU, *TEF1*, and *RPB2* genes are commonly used in *Ophiocordyceps* phylogenetics, as they provide reliable information across different taxonomic levels. In this study, a total of 42 new sequences were generated (10 ITS, 10 LSU, 12 *TEF1*, and 10 *RPB2*) (Table 1). Two strains of *Paraisariagracilis* (EFCC 3101 and EFCC 8572) in Ophiocordycipitaceae were used as outgroups. The combined dataset comprised 66 taxa, with multi-locus sequences totalling an alignment of 3,556 base pairs, including gaps (ITS: 707 bp, LSU: 959 bp, *TEF1*: 1,007 bp, and *RPB2*: 883 bp). The phylogenetic tree derived from maximum likelihood analysis, with bootstrap support values (MLB), is shown in Fig. 1. The nodes were also assessed using Bayesian posterior probabilities (BPP). Phylogenetic analyses revealed that *Ophiocordyceps* species from Diptera form a well-supported monophyletic group, here referred to as the ‘*O.dipterigena*’ complex. This clade includes four species identified as new in this study: *Ophiocordycepsfloriformis* (BBH 27634, BBH 51295), *O.muscae* (BCC 72871, BCC 73616, BCC 73607, NHJ12170.02, MRCIF71), *O.tabani* (BCC 39918, BCC 45127), and *O.thilosuensis* (BCC 46607, BCC 47494). Additionally, the strains BCC 22048, BCC 79225, BCC 78339, BCC 79871, and BCC 79872 clustered with the known *O.philippinensis* (LOD PF 4565), previously described from Diptera, and represent a new record for Thailand. Based on these findings, detailed species descriptions, including the morphological characteristics of *O.floriformis*, *O.muscae*, *O.philippinensis*, *O.tabani*, and *O.thilosuensis*, along with their dipteran hosts, are provided below. The morphological comparisons of *Ophiocordyceps* species associated with Diptera are shown in Table 2.

**Table 2.** Morphological comparisons of *Ophiocordyceps* species associated with Diptera.

Species	Host	Habitat	Origin	Stromata/Synnemata (mm)	Fertile part (mm)	Perithecia (µm)	Asci (µm)	Part-spores (µm)	Conidiogenous cells (µm)	Conidia (µm)	References
*O.dipterigena*	Diptera	On twig	Sri Lanka	Stromata: 5–10 × 1	Globose	–	–	10 × 1.5	–	–	Berkeley and Broome 1873
*O.discoideicapitata*	Diptera	Beneath a branch	Japan	Stromata: two, 2.5–3.5 × 0.7–1.2	Discoid, laterally conical, 3–4	Pyriform, 620–700 × 200–250	5–6 diam.	Cylindrical, truncated, 6–9 × 1	–	–	Kobayasi and Shimizu 1982
*O.floriformis*	Diptera (Asilidae, *Clephydroneura* sp.)	Underside of leaves of dicotyledonous plants	Thailand	Synnemata: several, clavate, 2–5 long, 50–100 μm wide	NA	NA	NA	NA	Hymenostilbe-like, phialidic, cylindrical, 10–20 × 2–4	Fusoid, 6–10 × 2–3	**This study**
*O.globiceps*	Diptera (Muscidae)	On grass stem	Thailand	Stromata: one, several, cinnamon to yellow, 4–8 × 0.5–1	Hemispherical to globoid, yellow, 1–1.5 long, 1–1.2 diam.	Ovoid to elongated pyriform, 538–663 × 182–247	373–454 × 5.7–8	Cylindrical to fusoid, 4–5.4 × 1.2–1.9	–	–	Xiao et al. 2019
*O.hemisphaerica*	Diptera (Muscidae)	On a twig	Brazil	Stromata: brown to greyish-brown, 12–20 × 0.8–1/ Synnemata: cylindrical, simple or branched, 6–12 × 0.5–1	Hemispherical, 1–1.2 long, 2–4 diam.	Obpyriform, slightly curved, 780–860 × 220–290	500–640 × 5–6	Cylindrical to unusually fusoid, 7–10 × 1–1.5	Hymenostilbe-like, phialidic, clavate, surface slightly rugose	Obovoid, 6.2–8.3 × 2.5–3.5	Hyde et al. 2016
*O.lacrimoidis*	Diptera (Muscidae)	On a twig	Brazil	Stromata: yellow, 4–5 × 1/ Synnemata: orange brown, 3 × 0.3	Discoid, pale to dark yellowish,1.2 long,1.8–2.2 diam.	Obpyriform, slightly curved, 650–700 × 200–250	350–450 × 5	Cylindrical, 8–14 × 2	Hymenostilbe-like, phialidic, clavate, surface roughened,	Lacrimoid, 4–5 × 3–5	Hyde et al. 2016
*O.muscae*	Diptera (*Muscadomestica*)	Underside of leaves of dicotyledonous plants	Thailand	Stromata: two, brownish-orange, 4–8 × 0.5–1.5/ Synnemata: solitary, brown to dark brown, 5–12 × 0.5–1.5	Hemispherical to globoid, orange yellow, 1–2 thick, 1.5–2 diam.	Ovoid to obclavate, 820–1100 × 320–400	Cylindrical, up to 720 long, 4–5 wide	Cylindrical to fusoid, 10–13 × 1.5–2	Hymenostilbe-like, phialidic, cylindrical, 12–20 × 3–4	Fusoid, 5–10 × 1.5–3	**This study**
*O.muscicola = C.muscicola*	Diptera (Muscidae)	Underside of leaves	Brazil	Stromata: two to six, rarely branched, 9–13 × 0.5–1/ Synnemata: 10 × 0.5	Discoid, 2–4 × 1–1.2	Pyriform, 850–920 × 230–300	550–700 × 5	Terminal cylindrical, intermediates fusoid, 8–10 × 1–2	Hymenostilbe-like, cylindrical, 11–14 × 2	Narrowly obovoid, 7–13 × 2–3	Möller 1901; Freire 2015
*O.philippinensis*	Diptera (Asilidae, *Asilus* sp.)	On a twig	Philippines	Stromata: several, ochre to brown, 5–6 × 1–2	Hemispherical to allantoid, 2–3 thick, 2–2.5 diam.	Ovate to elongate, pear-shaped, 236–256 × 98.5–113	140–160 × 3–4.5	–	–	–	Crous et al. 2023
*O.philippinensis*	Diptera (Asilidae, *Clephydroneura* sp.)	On a twig	Thailand	Stromata: several, brownish-orange, 5–8 × 1–1.5/ Synnemata: solitary, multiple, 4–20 × 0.5–1	Hemispherical to globoid, 2–2.5 thick, 3–4 diam.	Ovoid to obclavate, 720–1100 × 240–400	Cylindrical, up to 300 long, 4–6 wide	Cylindrical to fusoid, 10–12 × 1.5–2.5	Hymenostilbe-like, phialides cylindrical, 10–20 × 3–4	Fusiform, 5–10 × 2–3	**This study**
*O.tabani*	Diptera (Tabanidae, *Tabanus* sp.)	On a twig	Thailand	Stromata: two, brownish-orange, 4–10 × 1.5–2/ Synnemata: solitary, brown to dark brown, moderate orange, 5–10 × 0.5–2	Hemispherical to globoid, moderate orange, 2–3 long, 3–4 diam.	Ovoid to obclavate, 1000–1180 × 320–450	Cylindrical, 550–880 × 4–6	Cylindrical to fusoid, 10–14 × 1–2	Hymenostilbe-like, phialides cylindrical, 12–20 × 3–4	Fusoid, 5–10 × 3–4	**This study**
*O.thilosuensis*	Diptera (Tephritidae, *Anastrephaobliqua*; Stratiomyidae, Sarginae)	Underside of bamboo leaves	Thailand	Stromata: two, yellowish-white, 4–8 × 0.5–1.5/ Synnemata: multiple, yellowish- white, 3–10 × 0.5–1	Disc-shaped, upper surface slightly convex, yellowish white, 1–2 thick, 1–2.5 diam	Ovoid to obclavate, 700–1075 × 240–400	Cylindrical, 320–880 × 5–7	Cylindrical to fusoid, 6–12 × 1–2	Hymenostilbe-like, phialides cylindrical, 10–20 × 2–4	Obovoid, 5–8 × 2–3	**This study**

‘–’ information not provided in the original description. ‘NA’ indicates data not observed in this study.

### ﻿Taxonomy

#### ﻿ Ophiocordyceps floriformis

Taxon classificationFungiHypocrealesOphiocordycipitaceae

Tasanathai, Noisripoom & Luangsa-ard sp. nov.

0968E4C4-04D9-58CC-9422-1D1330031702

858732

Fig. 2

##### Etymology.

The name refers to the fungus’s outer appearance on the host, which resembles a small flower.

##### Typus.

Thailand • Nakhon Ratchasima Province, Khao Yai National Park, Kong Kaeo Waterfall, on robber fly (Asilidae, *Clephydroneura* sp.) attached to the underside of a dicotyledonous leaf of forest plant, 3 October 2022, J. Luangsa-ard, K. Tasanathai, S. Mongkolsamrit, A. Khonsanit, W. Noisripoom, D. Thanakitpipattana, N. Kobmoo, MY12948 (***holotype***BBH 51295).

**Figure 2.**
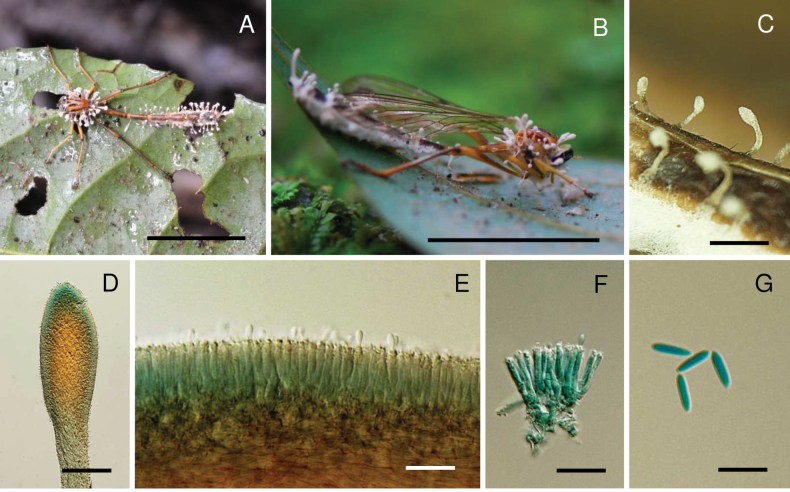
*Ophiocordycepsfloriformis*. **A, B.** Fungus on robber fly (Asilidae, *Clephydroneura* sp., Holotype BBH 51295); **C.** Synnemata; **D.** Head of synnema; **E, F.** Conidiogenous cells forming a hymenial layer; **G.** Conidia. Scale bars: 10 mm (**A, B**); 5 mm (**C**); 100 µm (**D**); 20 µm (**E, F**); 10 µm (**G**).

##### Description.

***Sexual morph***: Not observed. ***Asexual morph*: *Synnemata*** several, clavate, arising from the various parts of the head, thorax, and abdominal region of the host, cylindrical, unbranched, brown to dark brown at the base, light brown to grey towards the apex, 2–5 mm long, 50–100 μm wide, fertile region located at the terminal part of the synnemata. ***Conidiogenous cells***Hymenostilbe-like, phialidic, forming a hymenial layer. ***Phialides*** cylindrical with short crowded denticles, (10–)12–17(–20) × 2–4 μm (n = 35, 14.2 ± 3.1 × 2.6 ± 0.6 μm). ***Conidia*** hyaline smooth-walled, fusoid, 6–10 × 2–3 μm (n = 35, 8.1 ± 1.3 × 2.5 ± 0.5 μm).

##### Culture characteristics.

All isolates of *O.floriformis* were successfully obtained. However, no growth was observed on PDA during the primary isolation process, possibly due to its fastidious nature.

##### Host.

Robber fly (Asilidae, *Clephydroneura* sp.).

##### Habitat.

The specimen was found on the underside of a dicotyledonous leaf of forest plants.

##### Additional materials examined.

Thailand • Nakhon Ratchasima Province, Khao Yai National Park, Mo Singto Nature Trail, on robber fly (Asilidae, *Clephydroneura* sp.) attached to the underside of a dicotyledonous leaf of a forest plant, 20 July 2014, K. Tasanathai, P. Srikitikulchai, S. Mongkolsamrit, T. Chohmee, R. Ridkaew, MY4870 (paratype BBH 27634).

##### Notes.

*Ophiocordycepsfloriformis* exhibits a unique morphology by producing numerous short synnemata that emerge from the thorax and along the abdomen of its insect host. *Ophiocordycepsfloriformis* produces subglobose fertile structures at the terminals, which have a white conidial powder, resembling that of *O.buquetii*, a species commonly found on ants. The phialides of *O.floriformis* are Hymenostilbe-like, whereas in *O.buquetii*, the phialides are cylindrical with papillate ends (Mongkolsamrit et al. 2023). Based on the phylogenetic tree, *O.floriformis* belongs to the '*O.dipterigena*' complex, while *O.buquetii* is part of the '*O.australis*' complex. This species is fastidious, and no cultures could be obtained from the collected specimens. Hence, DNA was extracted from the synnemata of natural specimens.

#### ﻿ Ophiocordyceps muscae

Taxon classificationFungiHypocrealesOphiocordycipitaceae

Mongkolsamrit, Liangsiri, Thanakitpipattana & Luangsa-ard sp. nov.

224EB845-7BF3-54F4-97B6-4DF0E8EB5934

858733

Fig. 3

##### Etymology.

Named after the host genus, *Muscadomestica*.

##### Typus.

Thailand • Nakhon Ratchasima Province, Khao Yai National Park, Mo Singto Nature Trail, on housefly (*Muscadomestica*) attached to the underside of a dicotyledonous leaf of forest plant, 9 July 2014, K. Tasanathai, S. Mongkolsamrit, A. Khonsanit, W. Noisripoom, D. Thanakitpipattana, R. Somnuk, MY9689.01 (***holotype***BBH 41168, ex-type culture BCC 73616).

**Figure 3.**
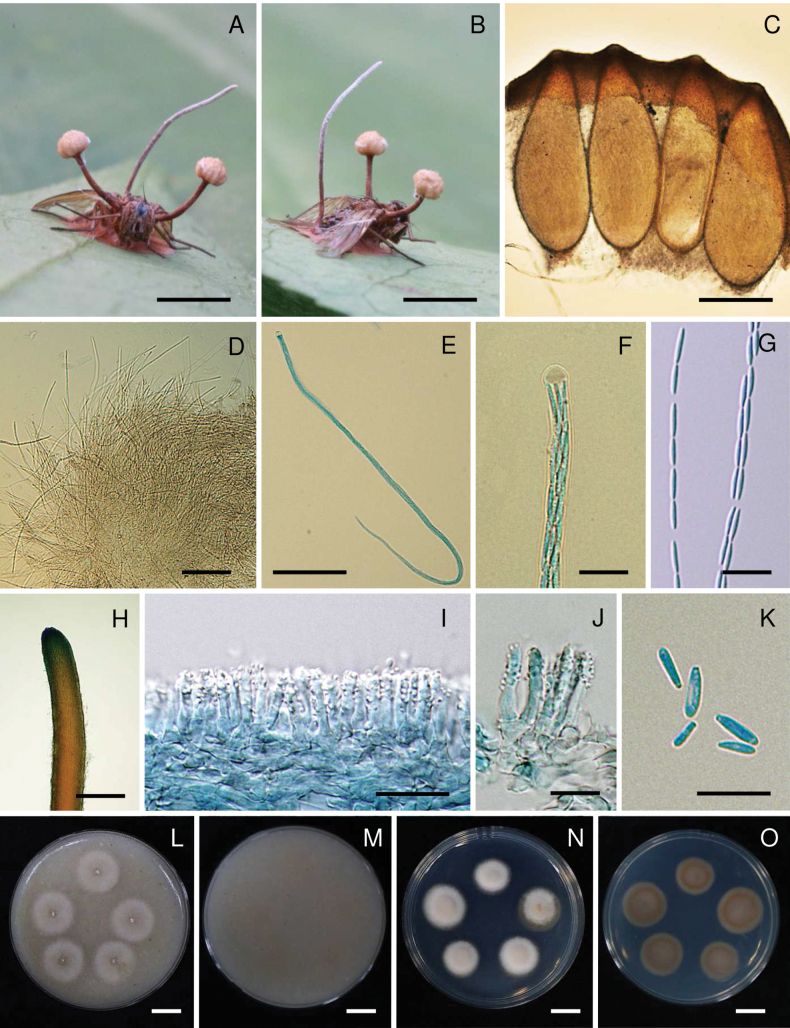
*Ophiocordycepsmuscae*. **A, B.** Fungus on housefly (*Muscadomestica*, Holotype BBH 41168); **C.** Perithecia; **D.** Asci; **E.** Ascus; **F.** Ascus cap; **G.** Part-spores; **H.** Synnema; **I, J.** Conidiogenous cells forming a hymenial layer; **K.** Conidia; **L, M.** Colonies on OA at 30 days (**L** obverse, **M** reverse) **N**, **O.** Colonies on PDA at 30 days (**N** obverse, **O** reverse). Scale bars: 5 mm (**A, B**); 350 µm (**C**); 100 µm (**D, E**); 10 µm (**F, J, K**); 20 µm (**G, I**); 1 mm (**H**); 10 mm (**L–O**).

##### Description.

***Stromata*** stipitate, usually two stromata arising from the thorax region of host, beneath the wings, capitate, unbranched. ***Stipes*** cylindrical, smooth, brownish-orange (165B), 4–8 mm long, 0.5–1.5 mm wide with a fertile apex. ***Sexual morph*: *Fertile heads*** hemispherical to globoid, upper surface slightly convex, moderate orange yellow (164B–C), located at the terminal part of stipes, 1–2 mm thick, 1.5–2 mm diam. ***Perithecia*** immersed, ovoid to obclavate, (820–)875–1020(–1100) × (320–)350–390(–400) μm (n = 20, 947.3 ± 71.2 × 367.5 ± 19 μm). ***Asci*** cylindrical, up to 720 μm long, 4–5 μm (n = 20, 4.4 ± 0.5 μm) wide, with cap 3–5 μm thick. ***Ascospores*** filiform, multi-septate, breaking into 64 part-spores, cylindrical to fusoid, 10–12(–13) × 1.5–2 μm (n = 50, 10.8 ± 0.8 × 1.8 ± 0.2 μm). ***Asexual morph*: *Synnemata*** usually arising from posterior abdomen region of host, solitary, cylindrical, unbranched, brown to dark brown at the base, light brown to grey towards the apex, 5–12 × 0.5–1.5 mm, fertile region located at about two-thirds of the synnema length. ***Conidiogenous cells***Hymenostilbe-like, phialidic, forming a hymenial layer. ***Phialides*** cylindrical with short crowded denticles, (12–)14–17.5(–20) × 3–4 μm (n = 30, 15.4 ± 1.9 × 3.3 ± 0.4 μm). ***Conidia*** hyaline smooth-walled, fusoid, (5–)6–8(–10) × 1.5–3 μm (n = 30, 6.8 ± 1.1 × 2.1 ± 0.2 μm).

##### Culture characteristics.

***Colonies*** on OA attaining a diam. of 7–10 mm in 30 days, mycelium sparse, white, reverse pale yellow (165D). ***Conidia*** and reproductive structures not observed. ***Colonies*** on PDA attaining a diam. of 7–10 mm in 30 days, high mycelium density, white, reverse pale yellow (165D). ***Conidia*** and reproductive structures not observed.

##### Host.

Housefly (Muscidae, *Muscadomestica*).

##### Habitat.

The specimens were found on the underside of a dicotyledonous leaf from a forest plant.

##### Additional materials examined.

Thailand • Nakhon Ratchasima Province, Khao Yai National Park, Mo Singto Nature Trail, on housefly (*Muscadomestica*) on the underside of a dicotyledonous leaf of a forest plant, 9 July 2014, K. Tasanathai, S. Mongkolsamrit, A. Khonsanit, W. Noisripoom, D. Thanakitpipattana, R. Somnuk, MY9670 (paratype BBH 38888, ex-paratype culture BCC 73607); • idem, 28 May 2014, K. Tasanathai, S. Mongkolsamrit, A. Khonsanit, W. Noisripoom, D. Thanakitpipattana, R. Somnuk, MY9584.01 (BBH 30660, culture BCC 48932); • idem, 30 April 2014, K. Tasanathai, P. Srikitikulchai, S. Mongkolsamrit, A. Khonsanit, W. Noisripoom, K. Sansatchanon, MY9568 (BBH 40602, culture BCC 72871); • Phetchabun Province, Nam Nao National Park, Headquarters Nature Trail, 3 October 2015, K. Tasanathai, S. Mongkolsamrit, W. Noisripoom, N. Kobmoo, R. Promharn, MY10980 (BBH 41221), MY10981 (BBH 41222); • Tak Province, Wat Phothi Khun Nature Trail, on housefly (*Muscadomestica*) on the underside of a dicotyledonous leaf of a forest plant, 20 August 2024, MY13633, K. Tasanathai, S. Mongkolsamrit, W. Noisripoom, K. Liangsiri.

##### Notes.

The two strains, NHJ12170.02 and MRCIF71, originally identified as *Ophiocordycepsdipterigena*, were collected from Thailand and have their sequences available in the NCBI database (Table 1). The sequence data for NHJ12170.02 were submitted by Luangsa-ard et al. (2011), while MRCIF71 was deposited by Aung and colleagues in 2008. However, based on our analysis and the phylogenetic results presented in Fig. 1, we found that these strains clustered with BCC 72871, BCC 73616, and BCC 73607, supporting their re-identification as *Ophiocordycepsmuscae*.

#### ﻿ Ophiocordyceps philippinensis

Taxon classificationFungiHypocrealesOphiocordycipitaceae

Piskorski, Kisło & Ruszk.-Mich., Persoonia 51: 345 (2023)

8BA2BBA2-60B7-5F28-B290-9380FF30DEB3

848172

Fig. 4

##### Note.

The description and illustrations are based on specimens of *O.philippinensis* collected in Thailand.

##### Description.

***Stromata*** stipitate, two to three stromata arising from the thorax region of host, beneath the wings, capitate, unbranched. ***Stipes*** cylindrical, smooth, brownish-orange (165B), 5–8 mm long, 1–1.5 mm wide with a fertile apex. ***Sexual morph*: *Fertile head*** hemispherical to globoid, upper surface slightly convex, moderate orange yellow (164C), located at the tip of stipes, 2–2.5 mm thick, 3–4 mm diam. ***Perithecia*** immersed, ovoid to obclavate, (720–)825–1017(–1100) × (240–)270–355(–400) μm (n = 30, 919.8 ± 97 × 313.4 ± 42 μm). ***Asci*** cylindrical, up to 300 μm long, 4–6 μm (n = 20, 5.3 ± 0.5) wide, with cap 4–6 μm thick. ***Ascospores*** filiform, multi-septate, breaking into 64 part-spores, cylindrical to fusoid, 10–12 × 1.5–2.5 μm (n = 50, 11 ± 1 × 2.1 ± 0.4 μm). ***Asexual morph***: ***Synnemata*** arising from head, thorax, abdomen regions of host, solitary, multiple, cylindrical, unbranched, brown to dark brown at the base, light brown to grey towards the apex, 4–20 × 0.5–1 mm, fertile part located at the two-thirds length of the synnemata. ***Conidiogenous cells***Hymenostilbe-like, phialidic, forming a hymenial layer. ***Phialides*** cylindrical with short crowded denticles, (10–)11.5–16.5(–20) × 3–3.5(–4) μm (n = 30, 14 ± 3 × 3.2 ± 0.4 μm). ***Conidia*** hyaline smooth-walled, fusiform, (5–)6 –8(–10) × 2–2.5(–3) μm (n = 30, 7.1 ± 1 × 2.2 ± 0.4 μm).

**Figure 4.**
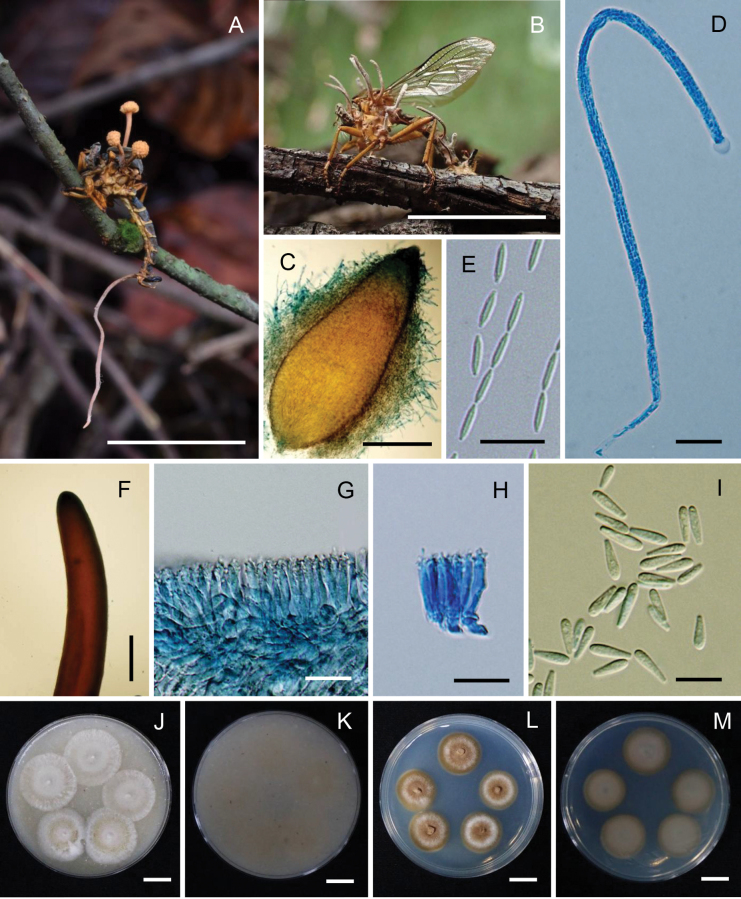
*Ophiocordycepsphilippinensis*. **A, B.** Fungus on a robber fly (Asilidae, *Clephydroneura* sp.); **C.** Perithecium; **D.** Ascus; **E.** Part-spores; **F.** Synnema; **G, H.** Conidiogenous cells forming a hymenial layer; **I.** Conidia; **J, K.** Colonies on OA at 30 days (**J** obverse, **K** reverse); **L**, **M.** Colonies on PDA at 30 days (**L** obverse, **M** reverse). Scale bars: 15 mm (**A, B**); 300 µm (**C**); 20 µm (**D, E**); 1 mm (**F**); 15 µm (**G, H**); 10 µm (**I**); 10 mm (**J–M**).

##### Culture characteristics.

***Colonies*** on OA attaining a diam. of 7–10 mm in 30 days, mycelium sparse, white, reverse pale yellow (165D). ***Conidia*** and reproductive structures not observed. ***Colonies*** on PDA attaining a diam. of 7–10 mm in 30 days, high mycelium density, white, reverse pale yellow (165D). ***Conidia*** and reproductive structures not observed.

##### Host.

Robber fly (Asilidae, *Clephydroneura* sp.)

##### Habitat.

Specimens were found on the twig of a tree in a forest.

##### Additional materials examined.

Thailand • Chaiyaphum Province, Phu Khiao Wildlife Sanctuary, Thung Ka Mang Nature Trail, on robber fly (Asilidae, *Clephydroneura* sp.) attached to the twig of a tree, 25 May 2006, K. Tasanathai, W. Chaygate, S. Mongkolsamrit, R. Ridkaew, B. Thongnuch, V. Sri-Indrasutdhi, MY1294 (BBH 17649, BCC 22048); • idem, 12 August 2015, S. Mongkolsamrit, A. Khonsanit, W. Noisripoom, D. Thanakitpipattana, N. Kobmoo, P. Srikitikulchai, S. Wongkanoun, R. Promharn, MY10790.01 (BBH 41274, BCC 79225), MY10790.02 (BBH 41274, BCC 78339); • Chiang Mai Province, Kanlayaniwatthana district, on robber fly (Asilidae, *Clephydroneura* sp.) attached to the twig of a tree, 23 November 2015, K. Tasanathai, S. Mongkolsamrit, D. Thanakitpipattana, W. Noisripoom, R. Promharn, P. Srikitikulchai, S. Wongkanoun, MY11132 (BBH 42752, BCC 79871), MY11134 (BBH 41232, BCC 79872).

##### Notes.

The multi-gene phylogenetic analysis (Fig. 1) revealed that the five Thai strains—BCC 79225, BCC 78339, BCC 79871, BCC 22048, and BCC 79872—clustered with *O.philippinensis*, previously reported from the Philippines (LOD PF 4565), with strong support (99% MLB/1 BPP). These species infect flies of the same family, Asilidae, but differ in their host genus. *Ophiocordycepsphilippinensis* collected in Thailand infects *Clephydroneura* sp., while *O.philippinensis* from the Philippines infects *Asilus* sp. A comparison of morphological features shows that the perithecia and asci of the Thai specimens are larger and longer than those of the Philippine specimens. The part-spores in the Thai specimens are mature, measuring 10–12 × 1.5–2.5 μm, whereas the part-spores in the Philippine specimens are immature (Table 2). Asexual morph reproductive structures, producing Hymenostilbe-like, were observed in the Thai specimen. Observations also revealed that the sexual and asexual morphs occur together on the same specimen, with synnemata emerging from the host’s abdominal region (Fig. 4A). If only the asexual morph is present, synnemata emerge from various parts of the host (Fig. 4B).

#### ﻿ Ophiocordyceps tabani

Taxon classificationFungiHypocrealesOphiocordycipitaceae

Mongkolsamrit, Liangsiri, Thanakitpipattana & Luangsa-ard sp. nov.

25D3C6ED-5B39-5370-A5C1-FD2B1DBC9288

858734

Fig. 5

##### Etymology.

Named after the host genus, *Tabanus*.

##### Typus.

Thailand • Nakhon Ratchasima Province, Khao Yai National Park, Mo Singto Nature Trail, on horsefly (Tabanidae, *Tabanus* sp.) attached to a twig of a forest tree, 4 November 2010, P. Srikitikulchai, S. Mongkolsamrit, A. Khonsanit, R. Somnuk, K. Sansatchanon, W. Noisripoom, MY6380.01 (BBH 30055, ex-type culture BCC 45127).

**Figure 5.**
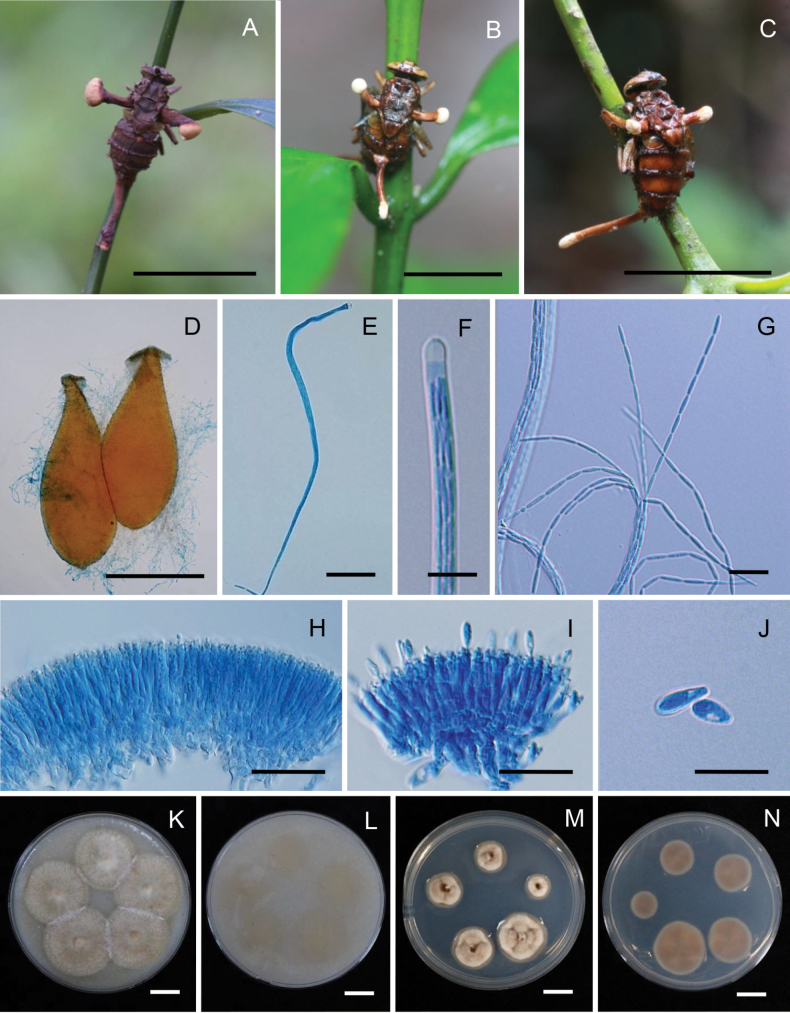
*Ophiocordycepstabani*. **A–C.** Fungus on a horsefly (Tabanidae, *Tabanus* sp., Holotype BBH 30055); **D.** Perithecia; **E.** Ascus; **F.** Ascus cap; **G.** Part-spores; **H, I.** Conidiogenous cells forming a hymenial layer; **J.** Conidia; **K, L.** Colonies on OA at 30 days (**K** obverse, **L** reverse); **M**, **N.** Colonies on PDA at 30 days (**M** obverse, **N** reverse). Scale bars: 15 mm (**A–C**); 500 µm (**D**); 100 µm (**E**); 10 µm (**F**); 20 µm (**G**); 15 µm (**H, I**); 10 µm (**J**); 10 mm (**K–N**).

##### Description.

***Stromata*** stipitate, usually two stromata arising from the thorax region of host, beneath the wings, capitate, unbranched. ***Stipes*** cylindrical, smooth, tough, brownish-orange (165 A–B), 4–10 mm long, 1.5–2 mm wide. ***Sexual morph*: *Fertile head*** hemispherical to globoid, upper surface slightly convex, moderate orange (167 B–C), located at the terminal part of stipes, 2–3 mm thick, 3–4 mm diam. ***Perithecia*** immersed, ovoid to obclavate, (1000–)1025–1148(–1180) × (320–)325–380(–450) μm (n = 15, 1087 ± 61.6 × 361 ± 43.2 μm). ***Asci*** cylindrical, (550–)636–870(–880) × (4–)4.5–6 μm (n = 30, 753.5 ± 116.8 × 5 ± 0.5 μm) with cap 3–6 μm thick. ***Ascospores*** filiform, multi-septate, breaking into 64 part-spores, cylindrical to fusoid, (10–)11–13(–14) × (1–)1.5–2 μm (n = 50, 11.8 ± 1.3 × 1.8 ± 0.3 μm). ***Asexual morph*: *Synnemata*** usually arising from posterior abdomen region of host, solitary, cylindrical, unbranched, uneven and rough when aged, brown to dark brown at the base, moderate orange at the apex, 5–10 × 0.5–2 mm, fertile part located at the terminal ends of the synnemata. ***Conidiogenous cells***Hymenostilbe-like, phialidic, forming a hymenial layer. ***Phialides*** cylindrical with short crowded denticles, (12–)13–17(–20) × 3–4 μm (n = 30, 15 ± 2.4 × 3 ± 0.2 μm). ***Conidia*** hyaline smooth-walled, fusoid, (5–)7–9(–10) × 3–4 μm (n = 30, 8 ± 1.2 × 3.3 ± 0.4 μm).

##### Culture characteristics.

***Colonies*** on OA attaining a diam. of 8–10 mm in 30 days, mycelium sparse, white, reverse pale yellow (165D). ***Conidia*** and reproductive structures not observed. ***Colonies*** on PDA attaining a diam. of 7–10 mm in 30 days, high mycelium density, white, reverse pale yellow (165D). ***Conidia*** and reproductive structures not observed.

##### Host.

Horsefly (Tabanidae, *Tabanus* sp.).

##### Habitat.

Specimens were found on the twig of a tree in a forest.

##### Additional materials examined.

• Nakhon Ratchasima Province, Khao Yai National Park, Mo Singto Nature Trail, horsefly (Tabanidae, *Tabanus* sp.) attached to a twig of a forest tree, 22 July 2009, K. Tasanathai, S. Mongkolsamrit, P. Srikitikulchai, R. Ridkaew, MY4999.01 (BBH 26791, Culture BCC 38243), MY4999.02 (paratype BBH 26791, ex-paratype culture BCC 39918); • idem, 30 June 2010, K. Tasanathai, P. Srikitikulchai, S. Mongkolsamrit, A. Khonsanit, R. Somnuk, K. Sansatchanon, MY6098.01, MY6098.02 (BBH 29675, Culture BCC 43730, Culture BCC 45070); • idem, 4 November 2010, P. Srikitikulchai, S. Mongkolsamrit, A. Khonsanit, R. Somnuk, K. Sansatchanon, W. Noisripoom, MY6380.01 (BBH 30055, Culture BCC 45127).

##### Notes.

*Ophiocordycepstabani* predominantly produces both sexual morphs and asexual morphs on the same specimen. Both *Ophiocordycepstabani* and *O.philippinensis* are commonly found attached to the twigs of trees. However, these two fungi differ in their hosts. *Ophiocordycepstabani* infects horseflies (Tabanidae, *Tabanus* sp.), while *O.philippinensis* infects *Clephydroneura* sp. and *Asilus* sp., both belonging to the Asilidae. The synnemata of *O.tabani* are tougher than those of other species. Notably, the reproductive structures of the asexual morph are located at the terminal ends of the synnemata, which often become uneven and rough with age. Among the specimens collected from the field, the hosts typically have no wings.

#### ﻿ Ophiocordyceps thilosuensis

Taxon classificationFungiHypocrealesOphiocordycipitaceae

Mongkolsamrit, Liangsiri, Thanakitpipattana & Luangsa-ard sp. nov.

D64084D6-84C3-5268-BE34-09CF97372DE3

858735

Fig. 6

##### Etymology.

Refers to the locality where the type specimen was found, Thi Lo Su Waterfall.

##### Typus.

Thailand • Tak Province, Umphang Wildlife Sanctuary, Thi Lo Su Waterfall, on fruit fly (Tephritidae, *Anastrephaobliqua*) attached to the underside of a bamboo leaf of a forest plant, 25 November 2010, K. Tasanathai, P. Srikitikulchai, A. Khonsanit, W. Noisripoom, K. Sansatchanon, MY6446.01 (**holotype**BBH 30265, ex-type culture BCC 46607).

**Figure 6.**
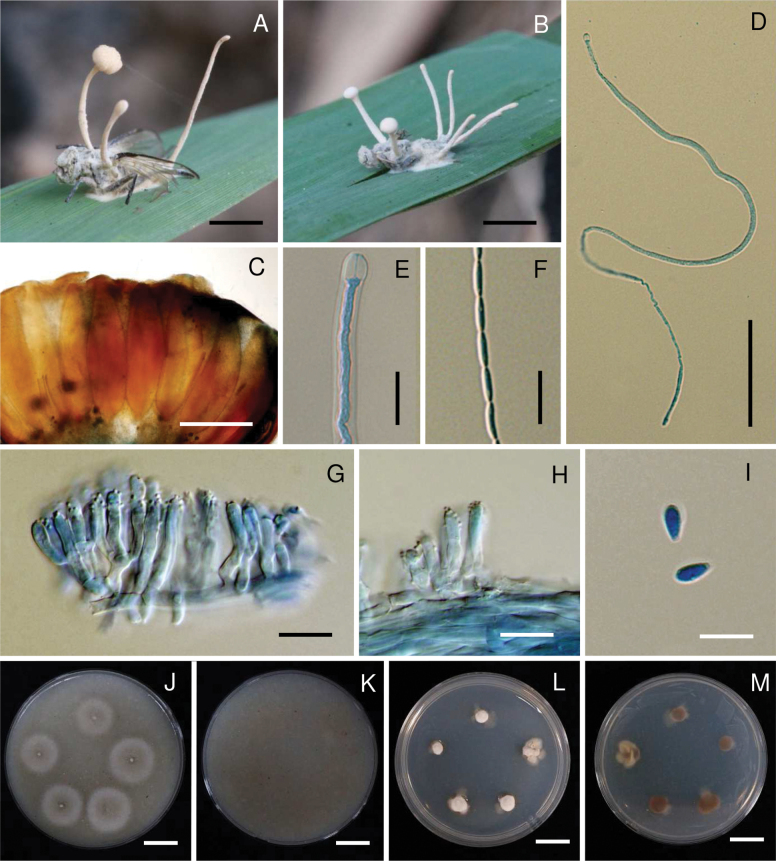
*Ophiocordycepsthilosuensis*. **A, B.** Fungus on a fruit fly (Tephritidae, *Anastrephaobliqua*, Holotype BBH 30265); **C.** Perithecia; **D.** Ascus; **E.** Ascus cap; **F.** Part-spores; **G, H.** Conidiogenous cells forming a hymenial layer; **I.** Conidia; **J, K.** Colonies on OA at 30 days (**J** obverse, **K** reverse); **L, M.** Colonies on PDA at 30 days (**L** obverse, **M** reverse). Scale bars: 3 mm (**A**); 4 mm (**B**); 500 µm (**C**); 80 µm (**D**); 10 µm (**E, F, G–I**); 10 mm (**J–M**).

##### Description.

The dead fly hosts were covered with sparse, yellowish-white hyphae. ***Stromata*** stipitate, two stromata arising from the thorax region of host, beneath the wings, capitate, unbranched. ***Stipes*** cylindrical, smooth, yellowish white (NN155A), 4–8 mm long, 0.5–1.5 mm wide. ***Sexual morph*: *Fertile heads*** disc-shaped, upper surface slightly convex, yellowish white, located at the tip of the stipes, 1–2 mm thick, 1–2.5 mm diam. ***Perithecia*** immersed, ovoid to obclavate, (700–)920–1065(–1075) × (240–)300–350(–400) μm (n = 30, 990.6 ± 72.3 × 325 ± 25.2 μm). ***Asci*** cylindrical, (320–)411–754(–880) × 5–7 μm (n = 30, 582.8 ± 171.4 × 6 ± 1 μm), with cap 3–6 μm thick. ***Ascospores*** filiform, multi-septate, breaking into 64 part-spores, cylindrical to fusoid, (6–)7.5–12 × (1–)1.5–2 μm (n = 50, 9.8 ± 2.1 × 1.6 ± 0.3 μm). ***Asexual morph*: *Synnemata*** arising from posterior abdomen region of host, solitary or multiple, cylindrical, unbranched, yellowish white, 3–10 × 0.5–1 mm, fertile part located at the two-thirds length of the synnemata. ***Conidiogenous cells***Hymenostilbe-like, phialidic, forming a hymenial layer. ***Phialides*** cylindrical with short crowded denticles, (10–)12–18(–20) × (2–)2.5–3.5(–4) μm (n = 30, 14.7 ± 3 × 3.1 ± 0.5 μm). ***Conidia*** hyaline smooth-walled, obovoid, (5–)5.5–7.5(–8) × 2–3 μm (n = 30, 6.5 ± 0.9 × 2.5 ± 0.5 μm).

##### Culture characteristics.

***Colonies*** on OA attaining a diam. of 7–10 mm in 30 days, mycelium sparse, white, reverse pale yellow (165D). ***Conidia*** and reproductive structures not observed. ***Colonies*** on PDA attaining a diam. of 7–10 mm in 30 days, high mycelium density, white, reverse pale yellow (165D). ***Conidia*** and reproductive structures not observed.

##### Host.

Fruit fly (Tephritidae, *Anastrephaobliqua*), soldier fly (Stratiomyidae, Sarginae).

##### Habitat.

Specimen was found on the underside of a bamboo leaf of a forest plant.

##### Additional materials examined.

Thailand • Tak Province, Umphang Wildlife Sanctuary, Thi Lo Su Waterfall, on fruit fly (Tephritidae, *Anastrephaobliqua*) attached to the underside of a bamboo leaf, 25 November 2010, K. Tasanathai, P. Srikitikulchai, A. Khonsanit, W. Noisripoom, K. Sansatchanon, MY6439 (paratype BBH 30099, ex-paratype culture BCC 47494), and MY6446.02 (BBH 30265, culture BCC 46608); MY6441 (BBH 30100, culture BCC 46606).

##### Notes.

*Ophiocordycepsthilosuensis* has been collected from a bamboo forest. This species exhibits unique morphological characteristics. The fly hosts are covered with sparse hyphae that are yellowish-white in colour. The stromata range from yellowish white. The fertile parts are disc-shaped and located at the terminal of the stipe. These characteristics, which resemble those of *Hevansianovoguineensis*, occur on spiders and can be found on the underside of dicotyledonous leaves in the forest (Mongkolsamrit et al. 2022).

## ﻿Discussion

During field surveys across various locations in Thailand, specimens exhibiting morphological features consistent with the broad concept of *Ophiocordycepsdipterigena* were discovered. These fungi, parasitic on flies, are characterised by yellow to orange-brown cylindrical stromata with fertile ascomata located at the tip. Although this species has been referenced in several studies, it lacks molecular data from the type locality, is limited by an incomplete original species description, and has no designated neotype (Salgado-Neto et al. 2018; Chhetri et al. 2020). Our phylogenetic analysis revealed that the specimens collected in Thailand as *O.dipterigena* sensu lato segregate into four new species: *O.floriformis*, *O.muscae*, *O.tabani*, and *O.thilosuensis*. Additionally, we report the first record of *O.philippinensis* in Thailand. This study also confirms the presence of several species belonging to the ‘*O.dipterigena*’ complex, including *O.globiceps*, *O.hemisphaerica*, *O.lacrimoidis*, and *O.dipterigena*, forming a strongly supported monophyletic group (Fig. 1). These species exhibit distinct characteristics consistent with the broader concept of *O.dipterigena*. Moreover, our study reveals that the ‘*O.dipterigena*’ complex is a subclade within the hymenostilboid clade, which includes other subclades such as the ‘*O.myrmecophila/irangiensis*’ complex, the ‘*O.australis*’ complex (associated with ants and wasps, Hymenoptera), and the '*O.nutans*' complex (associated with stink bugs, Hemiptera). Species in the ‘*O.dipterigena*’ complex share distinct morphological traits, including the production of fertile ascomata at the terminal end of a stipe with immersed perithecia and filiform ascospores that break into 64 part-spores. These features are characteristic of the related subclades and represent a shared derived character—a synapomorphy—of the hymenostilboid clade, as these fungi are primarily associated with the *Hymenostilbe* asexual morph (Khonsanit et al. 2019; Araújo et al. 2020; Khao-ngam et al. 2021). The newly discovered species play important ecological roles by parasitising various dipteran hosts, thus expanding our understanding of both the phylogenetic placement and ecological functions of these fungi, addressing gaps highlighted in previous studies.

*Ophiocordycepsphilippinensis*, originally discovered in the Philippines parasitising *Asilus* sp., has also been found in Thailand infecting *Clephydroneura* sp. Phylogenetic analysis shows that strains from both countries cluster together within a single well-supported clade. Although the host genera differ between the Philippines and Thailand, both specimens share similar morphological characteristics and ecological habits, such as growing on plant twigs. This indicates that *O.philippinensis* exhibits a broad host range within dipteran flies and a wide geographic distribution across Southeast Asia. These findings contribute to a better understanding of the biogeographic patterns of *Ophiocordyceps* species in the region, suggesting that while dispersal occurs across countries, localised evolutionary processes may influence genetic diversity. A similar pattern is observed in *O.buquetii* on ants (Araújo et al. 2020; Mongkolsamrit et al. 2023). Remarkably, *O.floriformis* is genetically closely related to *O.philippinensis*, forming a sister clade (Fig. 1). Both species parasitise *Clephydroneura* sp. (the same host of *O.philippinensis* in Thailand); however, they exhibit distinct morphological differences. *Ophiocordycepsfloriformis* produces short synnemata with white, powdery conidia at the tips, while *O.philippinensis* has cylindrical synnemata with greyish conidia scattered along the synnemata (Figs 2A–C, 4A, B). Furthermore, the ecological niche of *O.floriformis* differs, as it attaches to the underside of dicotyledonous plants, unlike *O.philippinensis*, which attaches to plant twigs. The two fungal species can be easily classified based on their morphological appearance, host, and habitat.

*Cordycepsmuscicola* was first described by Alfred Möller (1901) from specimens collected in Blumenau, Brazil. It is currently regarded as a synonym of *O.dipterigena* (Index Fungorum 2022). The original specimen of *C.muscicola* was lost, likely destroyed during World War II (Freire 2015). Freire and colleagues later revisited the type locality, collected new specimens, and designated a lectotype based on Möller’s protologue drawing, along with an epitype derived from the newly collected material. *Cordycepsmuscicola* exhibits distinct morphological features, including hyphae covering its host, multiple stromata emerging from the host body, and flattened fertile heads. Based on these traits, it has been proposed as a distinct species, separate from *O.dipterigena* (Freire 2015). Notably, its unique characteristics and ecology, found on the underside of leaves, closely resemble those of *O.thilosuensis*. However, the evolutionary relationship between *C.muscicola* and *O.thilosuensis* remains unclear due to the lack of molecular data for *C.muscicola*.

The newly discovered *Ophiocordyceps* species play important ecological roles by parasitising various dipteran hosts, including robber flies (*O.floriformis*), houseflies (*O.muscae*), horse flies (*O.tabani*), and fruit and soldier flies (*O.thilosuensis*). Several strains related to *O.philippinensis* were also found on *Clephydroneura* spp. These host-specific associations suggest a role in natural fly population control. Members of *Ophiocordyceps* and closely related genera such as *Hirsutella* have demonstrated strong potential for biological control of insect pests. For instance, *H.thompsonii* and *H.citriformis* have been used to manage coconut mites and citrus psyllids, respectively (Pérez-González et al. 2022; Jampameung et al. 2024). These findings support the potential application of these fungi in integrated pest management programmes. In addition, many entomopathogenic fungi produce bioactive compounds with antimicrobial or anticancer properties (Sharma and Sharma 2021; Shahbaz et al. 2024). Discovering new species may therefore increase the chance of finding novel compounds for agricultural or medical use in the future.

This study has some limitations, including reliance on herbarium specimens, which limited access to fresh material for further ecological and molecular analyses. Additionally, uneven sampling across regions may have affected a comprehensive understanding of species diversity and distribution. Nevertheless, our findings reveal a high diversity of *Ophiocordyceps* species and their associations with dipteran hosts. This indicates that these fungi are well adapted to local environments and play important ecological roles. Exploring their distribution enhances our knowledge of biodiversity patterns and co-evolutionary processes in tropical ecosystems. Future studies with more extensive new specimen sampling across regions will help to better understand the full diversity and ecological roles of these fungi.

## Supplementary Material

XML Treatment for Ophiocordyceps floriformis

XML Treatment for Ophiocordyceps muscae

XML Treatment for Ophiocordyceps philippinensis

XML Treatment for Ophiocordyceps tabani

XML Treatment for Ophiocordyceps thilosuensis

## ﻿Additional information

### Use of AI

No use of AI was reported.

### Author ORCIDs

Nattawut Rungjindamai https://orcid.org/0000-0002-2610-3214

Marc Stadler https://orcid.org/0000-0002-7284-8671

Jennifer Luangsa-ard https://orcid.org/0000-0001-6801-2145

### Data availability

All of the data that support the findings of this study are available in the main text.
